# Joint Association of Hydration Status and Sleep Quality with Type 2 Diabetes Mellitus in a Population-Based Study

**DOI:** 10.3390/nu18172929

**Published:** 2026-09-07

**Authors:** Miaolin Han, Yixi Zhang, Na Zhang, Changyou Zhao, Limin He, Jingyue Guo, Qiuyun Cheng, Guotian Lin, Fan Zhang

**Affiliations:** 1 Key Laboratory of Tropical Translational Medicine of Ministry of Education, School of Public Health, Hainan Medical University, 3 Xue Yuan Road, Longhua District, Haikou 571199, China; 2404304412@muhn.edu.cn (M.H.); 2304304025@muhn.edu.cn (Y.Z.); changyouzhao2025@muhn.edu.cn (C.Z.); helimin@muhn.edu.cn (L.H.); gjy522965464@163.com (J.G.); 2 Department of Nutrition and Food Hygiene, School of Public Health, Peking University, Beijing 100191, China; zhangna@bjmu.edu.cn; 3 Deyang Center for Disease Control and Prevention (Deyang Health Supervision Institute), No. 1, Section 1, Qianshan Street, Jingyang District, Deyang 618000, China; 4 Haikou Center for Disease Control and Prevention, 56 Yehai Avenue, Qiongshan District, Haikou 571100, China; 18289906762@163.com; 5 School of Tourism and Health, University of Sanya, 191 Xue Yuan Road, Jiyang District, Sanya 572022, China

**Keywords:** T2DM, hydration status, dehydration, sleep quality, interaction

## Abstract

**Objectives**: To examine the joint association of hydration status and sleep quality with prevalent type 2 diabetes mellitus (T2DM) and to quantify their additive and multiplicative interactions. **Methods**: This cross-sectional study included 4762 participants from the baseline Hainan Prospective Cohort Study. Logistic regression was used to estimate odds ratios (ORs) and 95% confidence intervals (CIs) for the four combinations of hydration status and sleep quality. Additive interaction was assessed with the relative excess risk due to interaction (RERI), the attributable proportion (AP), and the synergy index (S); multiplicative interaction was assessed with a product term. Predicted probabilities were estimated by g-computation. **Results**: The prevalence of T2DM was 8.13% (387/4762). Compared with non-dehydration and good sleep quality, the combination of dehydration and poor sleep quality was associated with substantially higher odds of prevalent T2DM (adjusted *OR* 9.92, *95% CI* 7.14 to 14.00). All three additive interaction measures were statistically significant (RERI 7.74, *95% CI* 4.99 to 10.50; AP 78.04%, *95% CI* 69.28 to 86.80; S 7.57, *95% CI* 3.49 to 16.42), as was the multiplicative interaction (OR 3.96, *95% CI* 2.30–6.90). On the absolute risk scale, the interaction contrast was 14.53 percentage points (*95% CI*: 11.01 to 18.34), with an attributable proportion of 80.5% (*95% CI*: 66.3 to 94.6) and a synergy index of 5.14 (*95% CI*: 2.94 to 17.25). The predicted probability of prevalent T2DM was highest in the combined dehydration and poor sleep group. **Conclusions**: The combination of dehydration and poor sleep quality was associated with higher odds of prevalent T2DM than either condition alone, and a statistically significant additive interaction was observed. Because these additive interaction measures were derived from odds ratios for a non-rare outcome, their absolute magnitude may be overestimated on the odds-ratio scale; the risk-difference-scale measures confirmed the interaction. Given the cross-sectional design, reverse causation cannot be excluded, and longitudinal data are needed to clarify the temporal sequence of these associations.

## 1. Introduction

Type 2 diabetes mellitus (T2DM) has become one of the most prevalent metabolic disorders worldwide. The high prevalence, disability burden, and substantial economic costs associated with T2DM pose substantial public health challenges [1]. According to the latest statistics from the International Diabetes Federation (IDF) [1,2], the number of adults living with diabetes worldwide has surpassed 500 million, accompanied by an increasing trend among younger populations. This figure is projected to continue rising over the coming decades. The pathogenesis of T2DM is complex [3,4], involving multidimensional interactions among genetic susceptibility, environmental factors, and lifestyle. Among these, lifestyle-related factors (such as dietary patterns, physical activity, and sleep patterns) have received increasing attention in T2DM research. A comprehensive understanding of these factors and their potential interactions may provide insights into T2DM prevention strategies [5,6].

Hydration status plays a crucial role in maintaining physiological homeostasis and has recently been investigated in relation to metabolic health [7]. Normal hydration levels are essential for blood circulation, renal filtration, cellular metabolism, and endocrine regulation [8]. Several biological mechanisms have been proposed to link chronic or subclinical dehydration with metabolic alterations: prolonged inadequate fluid intake may reduce blood volume and increase blood viscosity, potentially affecting insulin transport and sensitivity [9], while dehydration may also increase renal glucose reabsorption, which could be associated with impaired glucose regulation [10]. Epidemiological studies have reported associations between poor hydration status and metabolic conditions related to T2DM, such as obesity and hypertension [11]. However, evidence regarding the association between hydration status and T2DM, as well as its potential interaction with other factors such as sleep quality, remains limited.

Sleep quality, another critical lifestyle factor, has been extensively studied for its association with metabolic health. Normal sleep architecture and circadian regulation play important roles in maintaining hypothalamic–pituitary–adrenal (HPA) axis function and regulating appetite-related hormones, including leptin and ghrelin [12]. Conversely, sleep disorders, such as insomnia, sleep apnea-hypopnea syndrome, and abnormal sleep duration, have been associated with a higher risk of T2DM, with proposed mechanisms involving chronic inflammation, disruption of insulin signaling, and increased fat accumulation [13,14]. Although the independent association between sleep quality and T2DM has been widely investigated, whether hydration status and sleep quality are jointly associated with T2DM remains unclear.

Previous studies have suggested a bidirectional relationship between hydration status and sleep quality. The human body continuously loses water during sleep through respiration, skin evaporation, and other pathways. Insufficient fluid intake before bedtime or restricted nighttime drinking may contribute to morning dehydration, which has been associated with alterations in daytime cognitive function and metabolic regulation [15]. Conversely, sleep disturbances may be associated with hydration imbalance through alterations in renal concentrating ability and the secretion rhythm of antidiuretic hormone (ADH) [16]. This relationship raises the possibility that hydration status and sleep quality may be jointly associated with metabolic health.

However, the combined association of these two lifestyle-related factors with T2DM remains insufficiently explored. In particular, most previous studies have focused on the separate associations of hydration status and sleep quality with T2DM, whereas their joint association and interaction have rarely been quantitatively evaluated. Therefore, this study aimed to investigate the combined association of hydration status and sleep quality with prevalent T2DM and to provide epidemiological evidence for understanding their potential interaction.

## 2. Materials and Methods

### 2.1. Study Population

This cross-sectional study used baseline data from the Hainan Prospective Cohort Study of Chronic Diseases in the General Population (the Hainan Cohort), a population-based prospective cohort established to investigate the prevalence, incidence, and risk factors of major non-communicable diseases in Hainan Province, China [17]. In brief, between June 2018 and October 2020, community-dwelling residents aged 35–74 years were recruited from five areas of Hainan (Haikou, Sanya, Qionghai, Dongfang, and Qiongzhong), covering both urban and rural locations, using a multistage cluster random-sampling method. Participants were eligible if they were aged 35–74 years, had resided in Hainan for at least 5 years, had no severe physical disability or mental illness, and were able to complete the questionnaire interview, physical examination, and follow-up assessments. A total of 14,443 participants were enrolled at baseline (participation rate 90.1%) [17]. After written informed consent, baseline assessments comprised a face-to-face questionnaire administered by trained interviewers, a physical examination performed by trained physicians or nurses, and blood and urine collection with laboratory measurements. The questionnaire collected information on sociodemographic characteristics, medical and family history, lifestyle habits (smoking, alcohol drinking, tea drinking, dietary pattern, sleep quality, and physical activity), and other health-related factors. After an overnight fast of at least 10 h, fasting venous blood was drawn between 07:00 and 10:00, and midstream morning urine was collected on an empty stomach.

**Figure 1:**
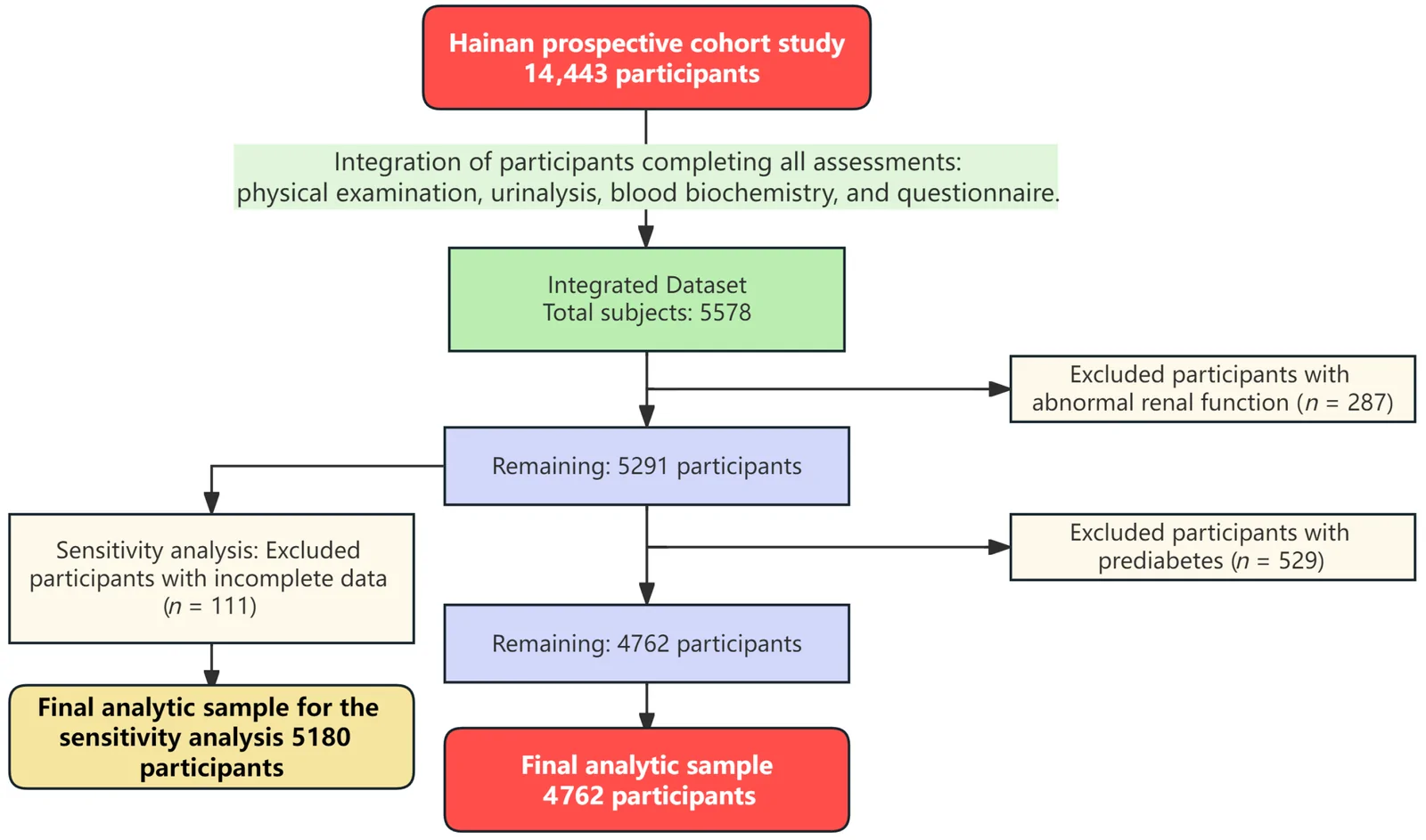
Data Cleaning Flowchart.

Enrollment in the cohort did not require completion of all baseline assessments; participants who completed at least one baseline assessment were enrolled [17]. For the present analysis, of the 14,443 participants enrolled at baseline, 5578 had completed all four assessments required here (the questionnaire, physical examination, blood biochemical testing, and urinalysis); the remaining participants completed only a subset of these assessments and were therefore not eligible. Participants with abnormal renal function (*n* = 287) and those with prediabetes (*n* = 529) were then excluded [18]. Impaired renal function directly affects urine concentration and thus the validity of USG as a hydration biomarker. Prediabetes was excluded as an analytical choice, because it is an intermediate dysglycemic state in which early metabolic disturbances—including possibly altered fluid intake—may already coexist, making it difficult in a cross-sectional design to determine whether altered hydration status or poor sleep precedes the development of diabetes or instead arises from the early dysglycemic process. After these exclusions, 4762 participants remained for the main analysis (Figure 1). Among the 529 participants with prediabetes, 418 with complete data were re-included in a sensitivity analysis (N = 5180); the remaining 111 were excluded because waist-to-hip ratio data (used to define central obesity) were missing.

### 2.2. Relevant Indicators and Definitions

#### 2.2.1. T2DM

Fasting venous blood samples (10 mL) were collected from participants. Fasting plasma glucose (FPG) levels were measured using 7600-110 (Hitachi, Tokyo, Japan) fully automated biochemical analyzer with corresponding reagent kits. T2DM was defined as an FPG level ≥ 7.0 mmol/L, in accordance with the diagnostic criteria of the American Diabetes Association [19]. Participants with a self-reported prior physician diagnosis of diabetes (previously diagnosed T2DM, *n* = 45) were excluded in a sensitivity analysis to address potential reverse causation.

#### 2.2.2. Chronic Kidney Disease

Serum creatinine (Scr) levels were measured using the Hitachi (Japan) 7600-110 fully automated biochemical analyzer with corresponding reagent kits. Estimated glomerular filtration rate (eGFR) was calculated using the Chronic Kidney Disease Epidemiology Collaboration (CKD-EPI) equation [20]:

$$e{GFR}_{female}=144*{\frac{Scr}{0.7}}^{-0.329}*0.993^{age}\;(Scr\leq 0.7\;mg/dL)$$

$$or=144*{\frac{Scr}{0.7}}^{-1.209}*0.993^{age}\;(Scr>0.7\;mg/dL)$$

$$e{GFR}_{male}=141*{\frac{Scr}{0.9}}^{-0.411}*0.993^{age}\;(Scr\leq 0.9\;mg/dL)$$

$$or=141*{\frac{Scr}{0.9}}^{-1.209}*0.993^{age}\;(Scr>0.9\;mg/dL)$$

eGFR was calculated using this equation; chronic kidney disease was defined as an eGFR < 60 mL/min/1.73 m^2^ [21].

#### 2.2.3. Central Obesity

Waist and hip circumferences were measured using a tape measure, and the waist-to-hip ratio (WHR) was calculated to assess central obesity. Central obesity was defined as WHR > 0.90 in men and >0.85 in women, according to previously established criteria [22].

#### 2.2.4. Hydration Status

First-void midstream urine samples were collected from participants. Urine specific gravity (USG) was measured using the COBIO Scan XL (Kobold, Germany) fully automated urine analyzer. Hydration status was classified according to USG values: adequate hydration (USG ≤ 1.010), intermediate hydration (1.010 < USG ≤ 1.025), and dehydration (USG > 1.025). For statistical analysis, the adequate and intermediate hydration categories were combined and classified as the non-dehydration reference group [23].

#### 2.2.5. Sleep Quality

Sleep quality was assessed using the Pittsburgh Sleep Quality Index (PSQI), which consists of seven components derived from 19 self-reported items. PSQI scores of 0 to 5, 6 to 10, 11 to 15, and 16 to 21 indicate good, fair, poor, and severely impaired sleep quality, respectively. In this study, participants with PSQI scores ≤ 10 were categorized as having good sleep quality, whereas those with scores > 10 were categorized as having poor sleep quality [24].

#### 2.2.6. Physical Activity Level

Physical activity was assessed using the International Physical Activity Questionnaire (IPAQ) Short Form. Metabolic equivalent task (MET) values were calculated according to questionnaire responses. Physical activity levels were categorized as low (<600 MET-min/week), moderate (600 to 3000 MET-min/week), and high (>3000 MET-min/week) [25].

#### 2.2.7. Smoking, Alcohol Drinking, and Tea Drinking

These lifestyle variables were derived from the baseline questionnaire. Participants who reported never smoking or having quit smoking for at least six months were classified as non-smokers (No); those who reported occasional or daily smoking, or who had quit for less than six months, were classified as smokers (Yes). Similarly, participants who reported never drinking alcohol or having abstained from alcohol for at least six months were classified as non-drinkers (No); current drinkers and those who had quit for less than six months were classified as drinkers (Yes). Tea drinking was defined as current regular tea drinking (at least one day per week); never or former tea drinkers were classified as non-tea drinkers (No).

#### 2.2.8. Chronic Disease

Chronic disease was defined as a self-reported physician diagnosis made at a township- or district-level hospital or a higher-level medical institution for any of the following conditions: coronary heart disease, stroke/transient ischemic attack, hypertension, hyperuricemia/gout, chronic bronchitis/emphysema/cor pulmonale, chronic obstructive pulmonary disease, or other chronic disease.

#### 2.2.9. Dietary Factors

Dietary intake was assessed using a food-frequency questionnaire (FFQ) administered as part of the baseline questionnaire, which recorded the frequency of consumption of major food groups. We initially attempted to derive dietary patterns using principal component analysis; however, the extracted components did not yield clearly interpretable dietary patterns. We therefore selected three dietary indicators for use as covariates in a sensitivity analysis: the consumption frequencies of livestock meat and meat products, fresh vegetables, and fresh fruits.

### 2.3. Statistical Analysis

Statistical analyses were performed using R version 4.4.3, with a two-sided *p* < 0.05 considered statistically significant. Categorical variables were summarized as frequencies and percentages, and continuous variables as mean ± SD. Between-group differences according to T2DM status were assessed using the Mann–Whitney U test (standardized Z statistic) for continuous variables and the Pearson chi-square test (without continuity correction) for categorical variables; percentages are within-category (row) proportions. To evaluate the discriminative performance of USG for assessing hydration status, ROC analysis was performed using urine osmolality as the reference standard. Binary logistic regression was used to estimate odds ratios (ORs) and 95% confidence intervals (CIs). Covariates were selected a priori on the basis of prior evidence and a causal framework as established risk factors for T2DM, rather than on the basis of univariate statistical significance, and included age (continuous), sex, BMI (continuous), central obesity, physical activity (light/moderate/vigorous), occupation (five categories), chronic disease, smoking, alcohol drinking, and tea drinking, see Table 1 for details. The joint association was evaluated by combining the two binary exposures into four groups, with non-dehydration (adequate/intermediate hydration) and good sleep quality as the reference group; both crude and adjusted ORs were reported.

Additive interaction was quantified by three measures—the relative excess risk due to interaction (RERI), the attributable proportion due to interaction (AP), and the synergy index (S), which were computed from the model containing the exposure product term and all covariates, where OR10, OR01, and OR11 denote the adjusted ORs for dehydration alone, poor sleep quality alone, and their combination, respectively, and these three measures were calculated as follows: RERI = OR11 − OR10 − OR01 + 1; AP = RERI/OR11; and S = (OR11 − 1)/[(OR10 − 1) + (OR01 − 1)] [26,27]. Because S is a strictly positive ratio, its *95% CI* was derived by the delta method on the log scale with back-transformation, while the CIs for RERI and AP were derived by the delta method on the original scale. Multiplicative interaction was assessed via the product term, reporting the interaction OR with its *95% CI* and a Wald test.

Marginal standardized predicted probabilities for the four groups were obtained by *g*-computation, with *95% CI*s from 1000 nonparametric bootstrap resamples (percentile method), for both crude and adjusted models. To quantify additive interaction on the absolute (risk difference) scale, we additionally derived the interaction contrast (IC), the attributable proportion due to interaction (AP), and the synergy index (S) from the marginal standardized predicted probabilities as IC = p11 − p10 − p01 + p00, AP = IC/(p11 − p00), and S = (p11 − p00)/[(p10 − p00) + (p01 − p00)], where p00, p10, p01, and p11 denote the adjusted predicted probabilities for non-dehydration with good sleep, dehydration with good sleep, non-dehydration with poor sleep, and dehydration with poor sleep, respectively. The *95% CI*s were estimated by the percentile method from the same 1000 bootstrap resamples. Five sensitivity analyses were conducted to assess the robustness of the findings: (1) re-including participants with prediabetes who had complete data (N = 5180); (2) excluding participants with previously diagnosed T2DM (N = 4717); (3) adjusting separately for BMI or central obesity; (4) further adjusting for dietary frequency (meat, vegetables, and fruit); and (5) modeling USG and PSQI as continuous standardized variables (per SD). Complete-case analysis was used, with no imputation. Except for the 111 participants with prediabetes excluded from the sensitivity analysis owing to missing waist-to-hip ratio data, no covariate values were missing in the analytic sample.

## 3. Results

### 3.1. Discriminatory Ability of Urine Specific Gravity

**Figure 2:**
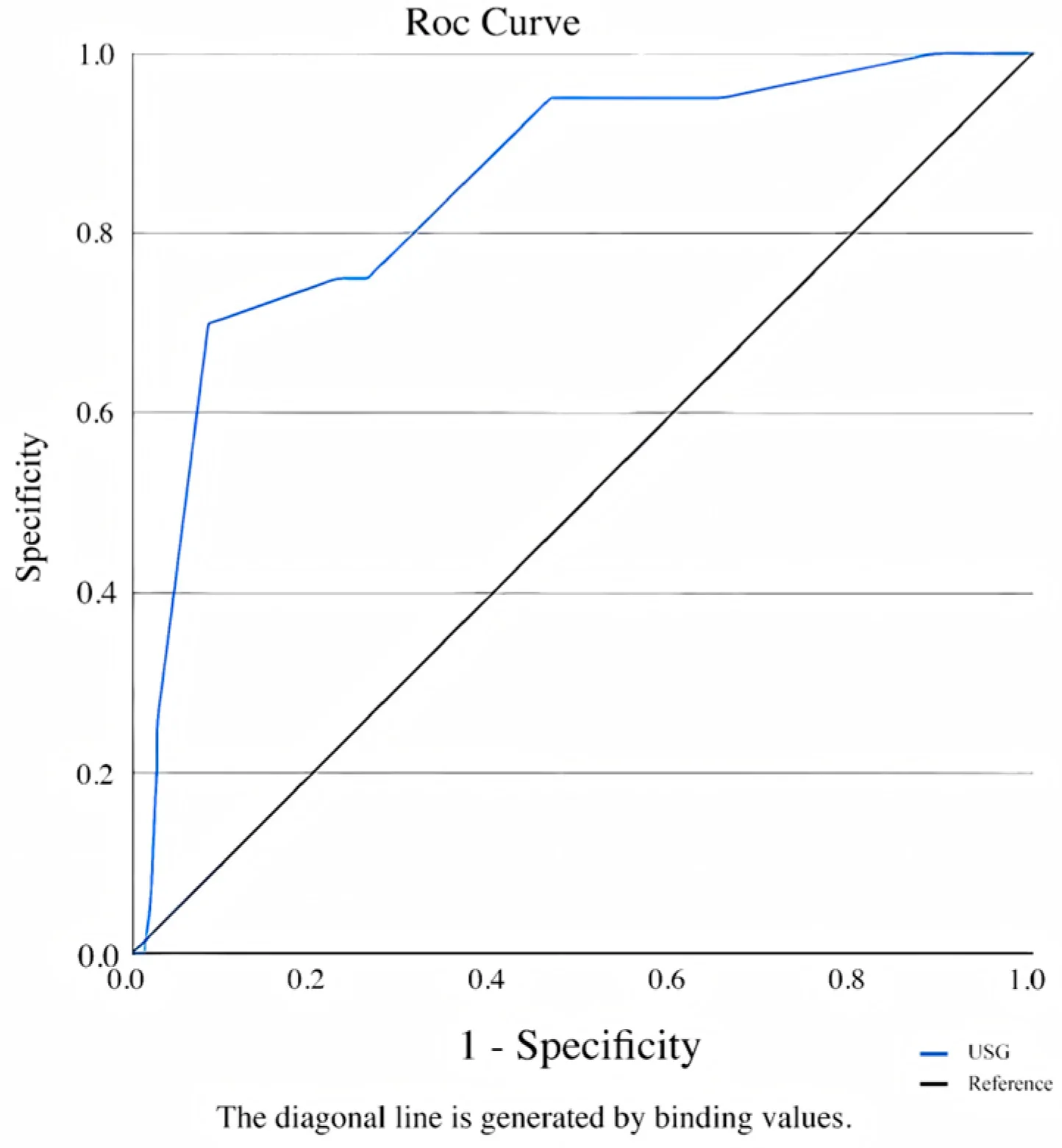
ROC Curve Assessing the Discriminative Ability of Urine Specific Gravity Using Urine Osmolality as the Reference Standard.

In the validation subsample of 375 older adults (166 men and 209 women, aged 65–79 years), using urine osmolality ≥ 800 mOsm/kg as the reference standard, the ROC analysis showed that the AUC for USG was 0.850 (*95% CI*: 0.763 to 0.938), with a standard error of 0.045 (*p* < 0.01), indicating good discriminatory performance, as shown in Figure 2.

### 3.2. Characteristics of the Study Participants

This study included a total of 4762 participants, comprising 2576 males and 2186 females. The mean age of participants was 45.87 ± 7.52 years, with a mean BMI of 23.42 ± 3.05. The T2DM prevalence was 8.13% (387/4762), as shown in Table 2.

### 3.3. Factors Associated with Prevalent T2DM

Significant differences between participants with and without T2DM were observed for age (*Z* = −13.27), BMI (*Z* = −6.62), sex ($\chi^{2}$ = 135.43), central obesity ($\chi^{2}$ = 81.03), hydration status ($\chi^{2}$ = 253.30), sleep quality ($\chi^{2}$ = 108.74), physical activity ($\chi^{2}$ = 16.06), occupation ($\chi^{2}$ = 13.03), chronic disease ($\chi^{2}$ = 51.85), smoking ($\chi^{2}$ = 70.81), alcohol drinking ($\chi^{2}$ = 9.88), and tea drinking ($\chi^{2}$ = 4.07) (*p* < 0.05 for occupation and tea drinking; *p* < 0.01 for all other variables). Participants with T2DM were older and had a higher BMI, and T2DM prevalence was higher among women and participants with central obesity, dehydration, poor sleep quality, unemployment, chronic disease, smoking, alcohol drinking, and no tea drinking (all *p* < 0.05), as shown in Table 2.

### 3.4. Analysis of the Joint Associations of Hydration Status and Sleep Quality with Prevalent T2DM

Unadjusted logistic regression analysis showed that individuals with both dehydration and poor sleep quality had higher odds of prevalent T2DM than those without dehydration and with good sleep quality (*OR* = 9.79, *95% CI*: 7.28 to 13.37). After adjustment for age, sex, BMI, central obesity, physical activity, occupation, chronic disease, smoking, alcohol drinking, and tea drinking, the joint presence of dehydration and poor sleep quality was associated with higher odds of prevalent T2DM compared with the reference group of non-dehydration and good sleep quality (adjusted *OR* = 9.92, *95% CI*: 7.14 to 14.00). See Table 3.

### 3.5. Analysis of the Additive Interaction Between Hydration Status and Sleep Quality

After adjusting for confounding factors, additive interaction between hydration status and sleep quality was evaluated using RERI, AP, and S. The RERI was 7.74 (*95% CI*: 4.99 to 10.50), the AP was 78.04% (*95% CI*: 69.28 to 86.80), and the S was 7.57 (*95% CI:* 3.49 to 16.42). All three measures were statistically significant, indicating a departure from additivity; the AP estimate suggested that the interaction component accounted for approximately 78% of the excess odds among participants with both exposures on the OR scale. See Table 4 for details. On the absolute (risk difference) scale, the interaction contrast (IC) was 14.53 percentage points (*95% CI*: 11.01 to 18.34), the attributable proportion (AP) was 80.5% (*95% CI*: 66.3 to 94.6), and the synergy index (S) was 5.14 (*95% CI*: 2.94 to 17.25), all statistically significant.

### 3.6. Analysis of the Multiplicative Interaction Between Hydration Status and Sleep Quality

**Figure 3:**
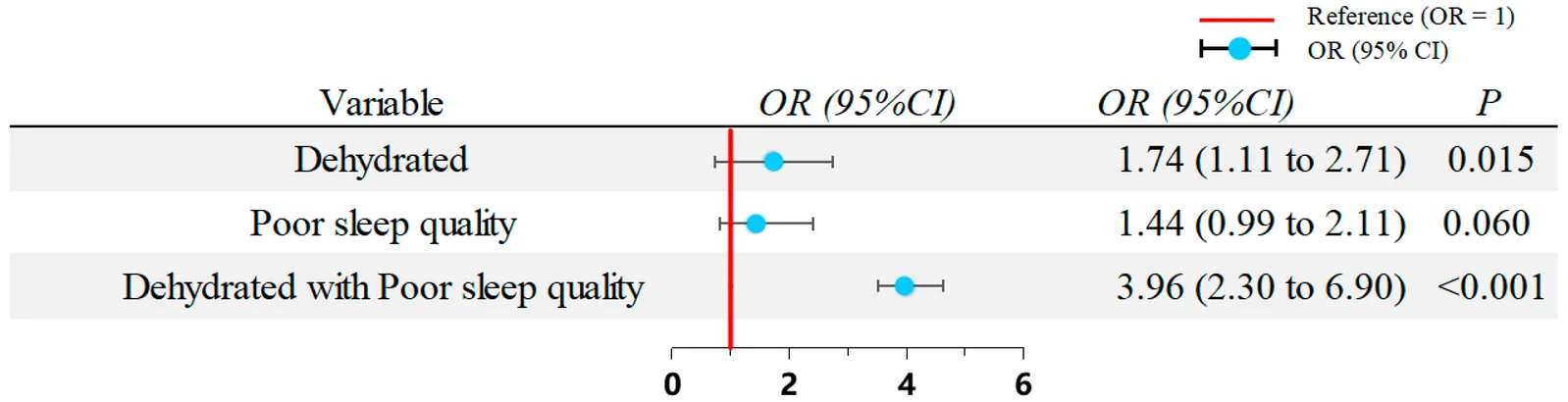
Multiplicative Interaction Between Hydration Status and Sleep Quality: Adjusted odds ratios (ORs) and 95% confidence intervals (CIs) were estimated from a multivariable logistic regression model containing hydration status, sleep quality, and their product term, adjusted for age, sex, BMI, central obesity, physical activity, occupation, chronic disease, smoking, alcohol drinking, and tea drinking. Dehydration and poor sleep quality showed a statistically significant multiplicative interaction (interaction OR = 3.96, *95% CI*: 2.30–6.90; *p* < 0.001). Abbreviations: OR, odds ratio; CI, confidence interval; T2DM, type 2 diabetes mellitus; BMI, body mass index.

After adjusting for confounding factors, the multivariate logistic regression model showed that individuals with dehydration had higher odds of prevalent T2DM compared with those without dehydration (*OR* = 1.74, *95% CI*: 1.11 to 2.71). The adjusted OR for poor versus good sleep quality was 1.41 (*95% CI*: 0.99 to 2.11). A statistically significant multiplicative interaction was observed between dehydration and poor sleep quality (interaction *OR* = 3.96, *95% CI*: 2.30 to 6.90). See Figure 3 for details.

### 3.7. Predicted Probability of Prevalent T2DM According to Hydration Status and Sleep Quality

After marginal standardization, the adjusted predicted probability of prevalent T2DM was 3.40% (*95% CI*: 2.60 to 4.30%) among participants without dehydration and with good sleep quality, compared with 21.50% (*95% CI*: 18.70 to 24.30%) among those with both dehydration and poor sleep quality. The corresponding crude predicted probabilities were 3.30% (*95% CI*: 2.50 to 4.10%) and 25.10% (*95% CI*: 22.20 to 28.00%), respectively. Predicted probabilities for all four exposure combinations are presented in Table 5.

### 3.8. Sensitivity Analyses

Sensitivity analyses showed that the main findings were generally consistent across alternative analytic samples and model specifications (Table 6). After including participants with prediabetes, the association between concurrent dehydration and poor sleep quality and prevalent T2DM remained strong (OR = 11.03, *95% CI*: 7.95 to 15.53). Similarly, after excluding participants with previously diagnosed T2DM, the corresponding OR was 9.90 (*95% CI*: 7.04 to 14.15). The estimates were also materially unchanged when BMI and central obesity were entered separately into the model and after further adjustment for dietary factors, with ORs for concurrent dehydration and poor sleep quality ranging from 9.57 to 10.15. Measures of additive interaction were broadly consistent across these sensitivity analyses (RERI: 7.42 to 8.49; AP: 76.9% to 80.9%; S: 6.49 to 10.05), and the multiplicative interaction ORs ranged from 3.52 to 4.77.

When USG and PSQI were analyzed as continuous standardized variables, higher USG and PSQI scores were each associated with greater odds of prevalent T2DM (OR per 1-SD increase = 1.60, *95% CI*: 1.41 to 1.82 for USG; OR = 1.81, *95% CI*: 1.60 to 2.06 for PSQI). A positive multiplicative interaction between continuous USG and PSQI scores was also observed (interaction OR = 1.58, *95% CI*: 1.39 to 1.80; *p* < 0.001) (Table 7).

## 4. Discussion

### 4.1. Association Between Combined Hydration Status and Sleep Quality Patterns and Prevalent T2DM

The prevalence of T2DM in this sample (8.13%) was close to the 8.6% reported at baseline for the same cohort [17], suggesting that the analytic sample had a similar T2DM prevalence to that of the overall baseline cohort. When hydration status and sleep quality were considered together, the combination of dehydration and poor sleep quality showed the strongest association with prevalent T2DM. Compared with the non-dehydrated, good-sleep group, in which T2DM prevalence was 3.32%, prevalence was 25.14% in the dehydrated, poor-sleep group, approximately 7.6 times as high. The crude odds of prevalent T2DM in this group were 9.79 times those of the reference group (*95% CI*, 7.28 to 13.37), which was higher than the corresponding estimates for either condition alone and was consistent with a stronger joint association. After adjustment for ten pre-specified confounders, the association persisted: participants who were both dehydrated and poor sleepers had 9.92 times the odds of prevalent T2DM compared with the reference group (*95% CI*, 7.14 to 14.00). These findings suggest that individuals with both characteristics may represent a subgroup with a higher prevalence of T2DM in metabolic screening settings.

Both poor sleep and poor hydration have been linked to abnormal glucose metabolism. Short or poor-quality sleep has been associated with a 20–40% higher risk of T2DM in prospective studies [28], while mild-to-moderate chronic dehydration has been associated with insulin resistance and impaired glucose regulation [29,30]. Most of this work, however, examined the two factors separately, and their combined association with T2DM has rarely been studied. Our analysis adds to this literature by considering hydration status and sleep quality together.

### 4.2. Interaction Between Hydration Status and Sleep Quality in Relation to Prevalent T2DM

This study used three measures—RERI, AP, and S—to quantify the additive interaction between hydration status and sleep quality. After adjusting for the same ten confounders, all three measures were statistically significant and mutually consistent: RERI was 7.74 (*95% CI*: 4.99 to 10.50), AP was 78.0% (*95% CI*: 69.28% to 86.80%), and S was 7.57 (*95% CI*: 3.49 to 16.42). These results indicate a departure from additivity, with the observed joint association exceeding that expected from the separate associations of dehydration and poor sleep quality on the OR scale; the AP estimate indicated that the interaction component accounted for approximately 78% of the excess odds among participants with both exposures. The multiplicative interaction was also significant (interaction OR = 3.96, *95% CI*: 2.30 to 6.90). These findings suggest a departure from additivity in the joint association of dehydration and poor sleep quality with prevalent T2DM. These interaction measures were derived from odds ratios. Because the outcome was not rare—particularly in the doubly exposed group, where the prevalence of T2DM reached 25.1%—the odds ratios may overestimate the corresponding prevalence ratios, and the additive interaction measures (RERI, AP, and S) may consequently be inflated in absolute magnitude. To provide an interpretable estimate on the absolute risk scale, we additionally computed the interaction directly from the marginal standardized predicted probabilities. On this risk-difference scale, the interaction contrast was 14.53 percentage points (*95% CI*: 11.01 to 18.34), the AP was 80.5% (*95% CI*: 66.3 to 94.6), and the S was 5.14 (*95% CI*: 2.94 to 17.25), indicating a substantial additive interaction that is directly interpretable as excess risk and is consistent with the OR-based measures.

Previous studies have applied interaction measures such as RERI, AP, and S to evaluate interactions involving metabolic and lifestyle-related factors [27,31,32]. Similar interaction patterns have been reported between obesity, physical activity, and metabolic syndrome-related outcomes [33]. Evaluating multiple lifestyle factors together may therefore provide additional information beyond single-factor analyses. Nevertheless, the interaction observed in the present study should be interpreted as an epidemiological association rather than as evidence of a causal synergistic effect. The joint-association and interaction estimates were also stable across the remaining sensitivity analyses—re-inclusion of participants with prediabetes, alternative handling of adiposity, further adjustment for dietary factors, and modeling of USG and PSQI as continuous variables—with RERI ranging from 7.42 to 8.49, AP from 76.9% to 80.9%, and S from 6.49 to 10.05 (Table 6 and Table 7).

### 4.3. Potential Mechanisms and Public Health Implications

At the mechanistic level, several biological pathways may help contextualize the observed association between concurrent dehydration and poor sleep quality and prevalent T2DM. Poor sleep quality has been associated with impaired glucose metabolism, with proposed mechanisms involving increased systemic inflammation, dysregulation of stress-related hormones, and reduced insulin sensitivity [34], whereas dehydration has been associated with alterations in glucose homeostasis potentially involving increased plasma osmolarity, activation of antidiuretic hormone pathways, and elevated cortisol levels [29]. The coexistence of impaired hydration status and poor sleep quality may therefore reflect overlapping physiological disturbances associated with metabolic dysregulation.

However, given the cross-sectional design, the observed associations should not be interpreted as evidence of causality, and reverse causation is an important concern. Individuals with undiagnosed or established T2DM may experience altered hydration status owing to osmotic diuresis, increased urinary glucose excretion, and polyuria [35,36], and diabetes-related nocturia, peripheral neuropathy, and psychological distress may impair sleep quality [37]. To address this concern, we repeated the analysis after excluding participants with previously diagnosed T2DM (N = 4717): the group with both dehydration and poor sleep quality still showed markedly higher odds of prevalent T2DM (adjusted OR = 9.90, *95% CI*: 7.04 to 14.15; RERI = 8.01, *95% CI*: 5.12 to 10.91), suggesting that the association was not fully explained by previously diagnosed diabetes-related dehydration or sleep disturbance, although reverse causation from undiagnosed or preclinical T2DM cannot be excluded. Future prospective studies with repeated measurements of hydration status, sleep characteristics, and glucose metabolism are needed to clarify the temporal sequence of these associations.

Furthermore, this study evaluated hydration status using urine specific gravity rather than urine osmolality. Although urine osmolality is commonly regarded as a reference indicator of hydration status, USG offers advantages of simplicity, lower cost, and feasibility for large-scale epidemiological investigations and primary healthcare settings [18,23], and the two measures are known to correlate strongly [38]. In a separate validation subsample of 375 older adults (aged 65–79 years), USG showed reasonable discrimination against urine osmolality ≥ 800 mOsm/kg (AUC 0.850); because this subsample was drawn from a different population and age group and USG was measured using a different instrument, these findings provide some support for the use of USG as a screening indicator, although their applicability to the present sample should be interpreted with caution.

### 4.4. Limitations

This study has several limitations. First, its cross-sectional design precludes causal inference, and reverse causation cannot be fully excluded despite the sensitivity analysis excluding previously diagnosed T2DM. In addition, information on glycemic control and glucose-lowering treatment was not collected at baseline, which further limits our ability to disentangle the direction of the association; these data will be collected in future follow-up assessments. Second, hydration status was assessed using a single morning urine specific gravity measurement rather than urine osmolality; because food and fluid intake and the timing of urine collection can influence USG, this may have resulted in misclassification of hydration status. Third, sleep quality, physical activity, smoking, alcohol drinking, and tea drinking were self-reported and are therefore subject to recall and reporting bias. Fourth, because the analytic sample was restricted to participants who completed all four assessments and participants with prediabetes were excluded from the primary analysis (including 111 who lacked waist-to-hip ratio data), the findings may be subject to selection bias and may not be fully generalizable to the entire baseline cohort; although this exclusion reduced reverse causation and outcome misclassification, it selected a more clearly normoglycemic sample, but the sensitivity analysis re-including participants with prediabetes who had complete data (N = 5180) yielded consistent results (Section 3.8).

## 5. Conclusions

This population-based cross-sectional study identified a strong association between combined dehydration and poor sleep quality and prevalent T2DM. Individuals with both conditions showed substantially higher odds of prevalent T2DM than those with either condition alone. Interaction analyses indicated a statistically significant additive interaction, together with a significant multiplicative interaction, although the absolute magnitude of the additive interaction may be overestimated because it was derived from odds ratios with a non-rare outcome. These findings suggest that the joint assessment of hydration status and sleep quality may help identify individuals with a higher prevalence of T2DM. However, causal relationships cannot be established owing to the cross-sectional design, and future longitudinal studies are warranted to clarify the temporal relationship between hydration status, sleep quality, and T2DM.

## Figures and Tables

**Table 1:** Coding of Variables.

Variable	Assignment
Gender	0 = Male, 1 = Female
Central obesity	0 = No, 1 = Yes
Hydration status	0 = Non-Dehydration, 1 = Dehydrated
Sleep quality	0 = Good sleep quality, 1 = Poor sleep quality
Physical activity	1 = Light, 2 = Moderate, 3 = Vigorous
Education	1 = Primary school or below, 2 = Secondary school or vocational school, 3 = College or above
Occupation	1 = Unemployed, 2 = Freelancer, 3 = Blue-collar worker, 4 = White-collar worker, 5 = Other
Chronic disease	0 = No, 1 = Yes
Smoking	0 = No, 1 = Yes
Alcohol drinking	0 = No, 1 = Yes
Tea drinking	0 = No, 1 = Yes

Note: Age and body mass index (BMI) were included as continuous covariates.

**Table 2:** Characteristics of Participants According to T2DM Status [*n* (%)].

Variable	Type 2 Diabetes Mellitus			$\chi^{2}$/*Z*-Value	*p*-Value
	No ^a^	Yes ^a^	Total ^b^		
Age ($\bar{X}\pm S$)	45.43 ± 7.33	50.84 ± 7.93	45.87 ± 7.52	−13.27	<0.001
Gender				135.43	<0.001
Male	2476 (96.12%)	100 (3.88%)	2576 (54.09%)		
Female	1899 (86.87%)	287 (13.13%)	2186 (45.91%)		
BMI	23.34 ± 3.03	24.41 ± 3.12	23.42 ± 3.05	−6.62	<0.001
Central obesity				81.03	<0.001
No	2432 (95.19%)	123 (4.81%)	2555 (53.65%)		
Yes	1943 (88.04%)	264 (11.96%)	2207 (46.35%)		
Hydration status				253.30	<0.001
Non-Dehydration	3088 (96.26%)	120 (3.74%)	3208 (67.37%)		
Dehydrated	1287 (82.82%)	267 (17.18%)	1554 (32.63%)		
Sleep quality				108.74	<0.001
Good sleep quality	2261 (96.05%)	93 (3.95%)	2354 (49.43%)		
Poor sleep quality	2114 (87.79%)	294 (12.21%)	2408 (50.57%)		
Physical activity				16.06	<0.001
Light	1585 (91.99%)	138 (8.01%)	1723 (36.18%)		
Moderate	1291 (89.72%)	148 (10.28%)	1439 (30.22%)		
Vigorous	1499 (93.69%)	101 (6.31%)	1600 (33.60%)		
Education				0.27	0.873
Primary school or below	571 (91.36%)	54 (8.64%)	625 (13.13%)		
Secondary school	2709 (91.99%)	236 (8.01%)	2945 (61.84%)		
College or above	1095 (91.86%)	97 (8.14%)	1192 (25.03%)		
Occupation				13.03	0.011
Unemployed	202 (89.78%)	23 (10.22%)	225 (4.73%)		
Freelancer	105 (97.22%)	3 (2.78%)	108 (2.27%)		
Blue-collar worker	2617 (92.64%)	208 (7.36%)	2825 (59.32%)		
White-collar worker	1218 (90.16%)	133 (9.84%)	1351 (28.37%)		
Other	233 (92.09%)	20 (7.91%)	253 (5.31%)		
Chronic disease				51.85	<0.001
No	3883 (92.94%)	295 (7.06%)	4178 (87.74%)		
Yes	492 (84.25%)	92 (15.75%)	584 (12.26%)		
Smoking				70.81	<0.001
No	3086 (94.11%)	193 (5.89%)	3279 (68.86%)		
Yes	1289 (86.92%)	194 (13.08%)	1483 (31.14%)		
Alcohol drinking				9.88	0.002
No	1770 (93.40%)	125 (6.60%)	1895 (39.79%)		
Yes	2605 (90.86%)	262 (9.14%)	2867 (60.21%)		
Tea drinking				4.07	0.044
No	1376 (90.71%)	141 (9.29%)	1517 (31.86%)		
Yes	2999 (92.42%)	246 (7.58%)	3245 (68.14%)		
Total	4375	387	4762		

Note: ^a^, Data are presented as numbers of participants with row percentages; ^b^, data are presented as numbers of participants with column percentages. Continuous variables are presented as mean ± SD. Age is expressed in years. Abbreviations: BMI, body mass index; T2DM, type 2 diabetes mellitus; Z, *Z*-value; χ^2^, chi-square value.

**Table 3:** Joint Associations of Hydration Status and Sleep Quality with Prevalent T2DM.

Hydration-Sleep Quality Groups	T2DM Cases	T2DM Prevalence	Crude OR (*95% CI*)	Adjusted OR (*95% CI*)
Non-Dehydrated with Good sleep quality	57	3.32%	1.00	1.00
Dehydrated with Good sleep quality	36	5.67%	1.75 (1.13 to 2.67)	1.74 (1.11 to 2.71)
Non-Dehydrated with Poor sleep quality	63	4.23%	1.29 (0.89 to 1.86)	1.44 (0.99 to 2.11)
Dehydrated with Poor sleep quality	231	25.14%	9.79 (7.28 to 13.37)	9.92 (7.14 to 14.00)

Note: The adjusted model was constructed to evaluate the association between combined hydration status and sleep quality categories and prevalent T2DM, adjusted for age, gender, BMI, central obesity, physical activity, occupation, chronic disease, smoking, alcohol drinking, and tea drinking.

**Table 4:** Additive Interaction Between Hydration Status and Sleep Quality.

Scale	Variable	Estimate	*95% CI*
Odds-ratio scale	RERI	7.74	4.99 to 10.50
	AP (%)	78.04	69.28 to 86.80
	S	7.57	3.49 to 16.42
Risk-difference scale	IC	14.53	11.01 to 18.34
	AP (%)	80.50	66.3 to 94.6
	S	5.14	2.94 to 17.25

Note: The adjusted model was adjusted for age, gender, BMI, central obesity, physical activity, occupation, chronic disease, smoking, alcohol drinking, and tea drinking. Abbreviations: RERI, relative excess risk due to interaction; AP, attributable proportion due to interaction; S, synergy index; CI, confidence interval.

**Table 5:** Marginal standardized predicted probabilities of prevalent T2DM according to hydration status and sleep quality.

Hydration Status and Sleep Quality	Crude Predicted Probability, % (*95% CI*)	Adjusted Predicted Probability, % (*95% CI*)
Non-Dehydrated with Good sleep quality	3.30 (2.50 to 4.10)	3.40 (2.60 to 4.30)
Dehydrated with Good sleep quality	5.70 (3.90 to 7.40)	5.60 (4.00 to 7.10)
Non-Dehydrated with Poor sleep quality	4.20 (3.20 to 5.20)	4.70 (3.70 to 5.90)
Dehydrated with Poor sleep quality	25.10 (22.20 to 28.00)	21.50 (18.70 to 24.30)

Notes: CI, confidence interval; T2DM, type 2 diabetes mellitus. Crude probabilities were estimated from a logistic regression model including hydration status, sleep quality, and their interaction. Adjusted probabilities were estimated from the multivariable logistic regression model using marginal standardization (*g*-computation). For each participant, the probability of prevalent T2DM was predicted under each of the four hypothetical combinations of hydration status and sleep quality while retaining the participant’s observed covariate values; the individual predicted probabilities were then averaged across the study population. The adjusted model included age, sex, BMI, central obesity, physical activity, occupation, chronic disease, smoking, alcohol drinking, and tea drinking. The *95% CI*s were estimated using 1000 bootstrap resamples.

**Table 6:** Sensitivity Analyses of the Joint Association and Interaction Between Hydration Status and Sleep Quality in Relation to Prevalent T2DM.

Sensitivity Analysis	Joint Associations			Additive Interaction			Multiplicative Interaction
	OR10 (*95% CI*)	OR01 (*95% CI*)	OR11 (*95% CI*)	RERI (*95% CI*)	AP, % (*95% CI*)	S (*95% CI*)	OR (*95% CI*)
Including participants with prediabetes	1.87 (1.20 to 2.89)	1.68 (1.15 to 2.45)	11.03 (7.95 to 15.53)	8.49 (5.45 to 11.52)	76.9 (68.3 to 85.5)	6.49 (3.45 to 12.19)	3.52 (2.06 to 6.08)
Excluding previously diagnosed T2DM	1.53 (0.94 to 2.45)	1.36 (0.91 to 2.03)	9.90 (7.04 to 14.15)	8.01 (5.12 to 10.91)	80.9 (72.5 to 89.4)	10.05 (3.62 to 27.84)	4.77 (2.68 to 8.65)
Adjusted for BMI but not central obesity	1.77 (1.13 to 2.75)	1.38 (0.95 to 2.02)	9.57 (6.90 to 13.47)	7.42 (4.79 to 10.04)	77.5 (68.4 to 86.5)	7.42 (3.37 to 16.32)	3.90 (2.27 to 6.79)
Adjusted for central obesity but not BMI	1.77 (1.13 to 2.76)	1.46 (1.00 to 2.14)	10.15 (7.31 to 14.30)	7.92 (5.10 to 10.73)	78.0 (69.3 to 86.7)	7.42 (3.50 to 15.72)	3.92 (2.28 to 6.82)
Further adjusted for dietary factors ^a^	1.77 (1.12 to 2.75)	1.43 (0.98 to 2.10)	10.12 (7.28 to 14.30)	7.93 (5.10 to 10.75)	78.3 (69.6 to 87.0)	7.61 (3.53 to 16.41)	4.00 (2.33 to 6.97)

Notes: OR, odds ratio; CI, confidence interval; RERI, relative excess risk due to interaction; AP, attributable proportion due to interaction; S, synergy index; BMI, body mass index; T2DM, type 2 diabetes mellitus. OR10 represents dehydration with good sleep quality; OR01 represents non-dehydration with poor sleep quality; and OR11 represents concurrent dehydration and poor sleep quality, with non-dehydration and good sleep quality as the reference group. All sensitivity models used the same covariate specification as the primary analysis except for the modification specified in each row. ^a^ Dietary factors included the consumption frequencies of livestock meat and meat products, fresh vegetables, and fresh fruits.

**Table 7:** Associations of Continuous USG and PSQI Scores With Prevalent T2DM in Sensitivity Analysis.

Exposure	Adjusted OR (*95% CI*)	*p* Value
USG, per 1-SD increase	1.60 (1.41 to 1.82)	<0.001
PSQI score, per 1-SD increase	1.81 (1.60 to 2.06)	<0.001
USG × PSQI interaction	1.58 (1.39 to 1.80)	<0.001

Notes: USG, urine specific gravity; PSQI, Pittsburgh Sleep Quality Index; SD, standard deviation; OR, odds ratio; CI, confidence interval. USG and PSQI scores were standardized before analysis; therefore, the ORs for the single-exposure estimates represent the change in the odds of prevalent T2DM per 1-SD increase.

## Data Availability

Data are available upon reasonable request from the corresponding author. Due to the need to protect patient privacy and ethical considerations, these data are not publicly available.

## Abbreviations

The following abbreviations are used in this manuscript:

T2DM	Type 2 Diabetes Mellitus
RERI	Relative Excess Risk of Interaction
AP	Attributable Proportion
S	Synergy Index
OR	Odds Ratio
CI	Confidence Interval
USG	Urine Specific Gravity
PSQI	Pittsburgh Sleep Quality Index
IDF	International Diabetes Federation
HPA	Hypothalamic–Pituitary–Adrenal
ADH	Antidiuretic Hormone
FPG	Fasting Plasma Glucose
Scr	Serum Creatinine
eGFR	estimated Glomerular Filtration Rate
CKD-EPI	Chronic Kidney Disease Epidemiology Collaboration
WHR	Waist-to-Hip Ratio
IPAQ	International Physical Activity Questionnaire
MET	Metabolic Equivalents of Task
ROC	Receiver Operating Characteristic
AUC	Area Under the Curve
BMI	Body Mass Index
